# Baseline and early lymphopenia predict for the risk of febrile neutropenia after chemotherapy

**DOI:** 10.1038/sj.bjc.6600724

**Published:** 2003-01-28

**Authors:** I Ray-Coquard, C Borg, Th Bachelot, C Sebban, I Philip, G Clapisson, A Le Cesne, P Biron, F Chauvin, J Y Blay

**Affiliations:** 1 Centre L. Bérard, 69008 Lyon, France; 2 Institut Gustave Roussy, 94805 Villejuif; 3 Hôpital Edouard Herriot, Hospices Civils de Lyon, Lyon 69903, France

**Keywords:** febrile neutropenia, lymphopenia, toxicity, chemotherapy, risk factors, risk model

## Abstract

A risk model for febrile neutropenia (FN) after conventional cytotoxic chemotherapy, based on early (day 5) lymphopenia and the dose of chemotherapy, has been described. A risk index based on parameters available at day 1 would be easier in daily practice. The objectives of this work were (1) to investigate a risk model for FN using only day 1 blood cell count and (2) to compare the day 1 and day 5 risk models. Three series of patients were used for the delineation and/or validation of these two risk models: (1) the exhaustive cohort of 950 patients treated in the Department of Medicine of the CLB in 1996 (CLB-1996 series), (2) the Elypse 1 series, a prospective series of 321 patients treated in community hospitals and regional cancer centres, and (3) a previously reported Elypse 0 series of 329 patients. Day 1 blood cell count was available in all three series, while day 5 blood cell count was available only in the Elypse 0 and 1 series. In the CLB-1996 series, 92 (9.7%) patients experienced FN; only chemotherapy dose and day 1 lymphopenia ⩽700 *μ*l^−1^ had an independent prognostic value for FN in multivariate analysis. In patients with both risk factors (‘high-risk group’), the incidence of FN was 44, 50 and 61% in the CLB-1996. Elypse 1 and 0 series, respectively, indicating that the ‘day 1’ risk model enables one to identify patients at high-risk for FN. Besides, the observed incidence of FN in the high-risk group of the ‘day 5’ model (i.e. patients with day 5 lymphopenia ⩽700 *μ*l^−1^ and receiving high-risk CT) was 45 and 69% in the Elypse 0 and 1 series, respectively. In the Elypse 1 and 0 series, 15 and 12% of all patients who experienced FN were in the high-risk group of the ‘day 1’ risk model as compared to 25 and 62% for the high-risk group of the ‘day 5’ risk model. Both day 1 and day 5 lymphopenia are associated with an increased risk of FN in patients treated with chemotherapy. The ‘day 1’ model identifies a small population of patients at high risk for FN, but has a lower sensitivity than the day 5 model.

Febrile grade four (PMN⩽500 *μ*l^−1^) neutropenia (FN) is a frequent complication of cancer chemotherapy, which causes death in 4–21% of patients in large studies (The International Antimicrobial Therapy Group, 1978; Bodey, 1986; Pizzo *et al*, 1987; Talcott *et al*, 1992; Klastersky, 1993; Bodey *et al*, 1996; Segal *et al*, 2001). The risk of FN after administration of chemotherapy not only depends on the type and doses of drugs administered, but also on individual risk factors for each patient, such as performance status (PS), coexisting infections or underlying immunosuppression (Bodey, 1986; Klastersky, 1993; Bodey *et al*, 1996; Segal *et al*, 2001). Hence, even after the administration of a dose-intensive chemotherapy regimen, 30–50% of the patients will not experience FN, while conversely, FN will occur in 2–15% of the patients who receive chemotherapy regimens with conventional doses of drugs (Miser *et al*, 1987; Tannock *et al*, 1988; Velasquez *et al*, 1988; Coiffier *et al*, 1989; Elias *et al*, 1989; Nichols *et al*, 1991).

The identification of simple and readily available clinical and biological risk factors for FN after the administration of cytotoxic chemotherapy is an important goal for the selection of candidate patients for prophylactic measures to FN, in particular administration of granulocyte growth factors (The American Society of Clinical Oncology, 1994; Blay *et al*, 1995; Boogaerts *et al*, 1995; Bennett *et al*, 1999). We previously reported that the occurrence of an early lymphopenia (⩽700 *μ*l^−1^) at day 5 following the administration of chemotherapy was an independent risk factor for FN along with the type of chemotherapy regimen in four series of patients treated in comprehensive cancer centres (Blay *et al*, 1996). In this prognostic model, a high-risk subgroup of patients with both risk factors (i.e. receiving ‘high-risk’ chemotherapy and with a day 5 lymphocyte count ⩽700 *μ*l^−1^) was identified, with an observed incidence of FN of 69%. This subgroup includes 62% of patients who experienced FN in this previous study (Blay *et al*, 1996). This model is currently used as inclusion criteria in a prospective randomised study comparing the use of G-CSF in primary or secondary prophylaxis. However, the use of day 5 lymphocyte count for the quantification of the risk of FN was found to be not optimally practical for outpatient care, since the patient is identified at high risk after having left the hospital. Although day 1 lymphocytes count was not significantly correlated to the risk of FN in the previous study, the observed incidence of FN in this subgroup was >40%, suggesting that it may have been missed because of a lack of power of the study (Blay *et al*, 1996). Supporting this hypothesis, baseline day 1 lymphopenia was found to be an independent risk factor for other haematological toxicities, in particular severe anaemia (Ray-Coquard *et al*, 1999), severe thrombopenia (Blay *et al*, 1998) and early death (Ray-Coquard *et al*, 2001) after cytotoxic chemotherapy.

To address this issue, a risk model for FN based on day 1 lymphocyte count was delineated in a large series of 950 patients (CLB-1996). The prognostic value of the two risk models for febrile neutropenia, relying respectively on day 1 and day 5 haematological parameters, was then tested and compared in two validation series.

## PATIENTS AND METHODS

### Selection criteria for patients

Selection criteria for patients in all series were identical and as follows: age above 17 years, negative human immunodeficiency virus (HIV) serology in patients with non Hodgkin's lymphoma, and chemotherapy regimens administered sequentially, that is every 8 or more days. All patients treated with cytotoxic chemotherapy were included, regardless of the number of previous courses. Exclusion criteria were: a diagnosis of low-grade lymphoma or leukaemia because of the possible contamination of peripheral blood by malignant lymphocytes, administration of cytokines or granulocyte growth factors during or after chemotherapy, and high-dose chemotherapy regimen requiring bone marrow or peripheral-blood stem-cell reinjection. Information about histology, primary tumour site, chemotherapy regimen, sex, age, PS, blood cell count on day 1 (d1) just before the administration of chemotherapy and on day 5, and FN following the studied chemotherapy course were collected. In the CLB-1996 series, the history of FN was not available for the majority of the patients, and therefore not collected. This information is available for the Elypse 1 series only. Each patient was analysed for only one course of chemotherapy. No prophylactic antibiotics were used in any series. Patients' characteristics are indicated in Table 1

**Table 1:** Clinical characteristics and correlation with febrile neutropenia

	**CLB-1996**	**Elypse 1**	**Elypse 0**									
	***N* (%)**	**FN**	***N* (%)**	**FN**	***N* (%)**	**FN**						
	**950**	***N***	**(%)**	***P***	**321**	***N***	**(%)**	***P***	**329**	***N***	**(%)**	***P***
Median age (range)	55 (18–86)				58 (18–86)				46 (18–80)			
Age (years)				***0.34***				**0.02**				**0.03**
⩽60	575 (60)	58	(10)		134	4	(3)		238 (72)	73	(31)	
>60	375 (40)	34	(9)		175	16	(9)		91 (28)	17	(19)	
												
Gender				**0.01**				***0.88***				***0.32***
F	514 (54)	61	(12)		194 (62)	12	(6)		169 (5116)	50	(34)	
M	436 (46)	31	(7)		121 (38)	8	(7)		49	40	(25)	
												
Diagnostic				**<0.001^a^**				**<0.001**				<0.001^a^
All carcinoma	734 (77)	58	(8)		281	12	(4)		138 (42)	15	(4)	
Breast	224 (24)	28	(12)		106 (33)	2	(2)		48 (15)	3	(6)	
Colon-rectum	91 (9)	1	(1)		55 (17)	0	(0)		10 (3)	0	(0)	
Ovary	86 (9)	13	(15)		36 (11)	3	(9)		10 (3)	0	(0)	
Head and neck	97 (10)	4	(4)		21 (7)	2	(10)		12 (4)	0	(0)	
Lung cancer	55 (6)	4	(8)		9 (3)	1	(11)		11 (3)	3	(27)	
Lymphoma/myeloma	72 (8)	16	(22)		21 (7)	4	(20)		115 (35)	52	(45)	
Sarcomas	64 (7)	10	(15)		15 (5)	4	(27)		48 (15)	(20)	(42)	
Germ cells tumor	29 (3)	4	(13)		7 (2)	2	(29)		28 (8)	3	(11)	
Others	232 (24)	12	(5)		51 (16)	2	(4)		48 (15)	2	(4)	
												
High-risk CT				**0.003**				**0.001**				**0.001**
Yes	89 (10)	17	(19)		24 (8)	7	(29)		142 (43)	70	(49)	
No	861 (90)	75	(9)		291 (92)	13	(4)		187 (572)	20	(11)	
												
PS				**0.03**				**0.04**				*0.07*^b^
0–1	801 (84)	71	(9)		251 (79)	14	(6)		98 (87)^b^	23^b^	(22)	
>1	149 (16)	21	(14)		40 (12)	6	(15)		14 (13)^b^	6^b^	(42)	
												
No. of previous courses of CT				*0.09*				***0.49***				***0.99***
0	450 (47)	47	(10)		97 (30)	9	(9)		106 (32)	29	(27)	
>1	600 (53)	45	(7)		224 (70)	11	(5)		223 (8)	61	(27)	
												
Metastases				**0.09**				***0.33***				***0.33***
Yes	121 (13)	17	(14)		95 (46)	4	(4)		95 (46)	4	(4)	
No	829 (87)	75	(9)		113 (54)	9	(8)		113 (54)	9	(8)	
												
Bone marrow metastases								0.69				0.17
Yes	NA	NA	NA		16	2	11		21	11	(52)	
No	NA	NA	NA		132	11	8		67	24	(36)	
												
Comorbidity				***0.52***								
Yes	163 (17)	16	(10)		NA	NA	NA		NA	NA	NA	
No	787 (83%)	76	(9.7)		NA	NA	NA		NA	NA	NA	
												
Previous FN								**0.005**				
Yes	NA	NA	NA		7	2	29		NA	NA	NA	
No	NA	NA	NA		278	13	4.7		NA	NA	NA	

a Lymphoma or myeloma *vs* sarcoma *vs* carcinomas and others.

b PS at the date of the course was available in 112 of the 329 patients. *P* value refers to *χ*^2^ test for association between risk factor and FN. FN=febrile neutropenia; NA=not available.

and Table 2

**Table 2:** Blood cell count and FN

	**CLB-1996 No. of**	**FN**		**Elypse 1 No. of**	**FN**		**Elypse 0 No. of**	**FN**				
	**patients (%)**	***N***	**(%)**	***χ*^2^ *P***	**patients (%)**	***N***	**(%)**	***χ*^2^ *P***	**patients (%)^a^**	***N***	**(%)**	***χ*^2^ *P***
Day 1 lymphocyte count				**0.05**				0.18				0.08
⩽700 *μ*l^−1^	204 (22)	27	(14)		36 (11)	4	(11)		227 (82)	48	(21)	
>700 *μ*l^−1^	746 (78)	65	(8)		277 (89)	16	(6)		49 (18)	16	(33)	
												
Day 5 lymphocyte count				NA				**0.004**				**<0.001**
⩽700 *μ*l^−1^	NA	NA			57 (21)	9	(16)		146 (44)	71	(49)	
>700 *μ*l^−1^	NA	NA			253 (79)	11	(4)		183 (56)	19	(10)	
												
Day 1 PMN count				0.41				0.67				0.16
⩽1500 *μ*l^−1^	19 (2)	2	(9)		18 (8)	1	(6)		23 (8)	8	(35)	
>1500 *μ*l^−1^	931 (98)	90	(9)		295 (92)	19	(6)		253 (92)	56	(22)	
												
Day 5 PMN count				NA				0.45				0.07
⩽1500 *μ*l^−1^	NA	NA			9 (3)	1	(5)		16 (5)	7	(44)	
>1500 *μ*l^−1^	NA	NA			301 (97)	19	(6)		257 (95)	59	(23)	
												
Day 1 platelet count				0.13				0.61				**<0.001**
<150 000 *μ*l^−1^	89 (9)	13	(14)		14 (5)	1	(7)		23 (9)	17	(71)	
>150 000 *μ*l^−1^	861 (91)	79	(9)		299 (95)	19	(6)		253 (91)	47	(19)	
												
Day 1 haemoglobin count				0.22				0.10				**0.04**
<12 g dl^−1^	448 (47)	49	(11)		161 (51)	7	(4)		160 (58)	44	(27)	
>12 g dl^−1^	502 (53)	43	(9)		154 (49)	13	(8)		117 (42)	20	(17)	

a Missing data in 56 patients. FN=febrile neutropenia, NA=not available. *P* value refers to *χ*^2^ test for association between risk factor and FN.

.

### Description of the patients

#### CLB-1996 series

This series is the exhaustive cohort of patients treated with conventional chemotherapy regimens in the Department of Medicine of the Centre Léon Bérard in 1996 who matched the selection criteria (Table 1). There were 3223 chemotherapy courses given to 1116 patients. Patients previously treated outside the centre were eligible. Patients with a missing blood cell count at day 1 (*n*=65) and receiving G-CSF or GM-CSF (*n*=101) after the course were excluded. Therefore, 950 of the 1116 patients (85%) were eligible and analysed.

#### Elypse 1 series

The prospective cohort (Elypse 1) is a multicentric prospective series of 321 patients treated with conventional chemotherapy in both general community hospital (*n*=13) and regional cancer centres (*n*=5): 84 patients (26%) were treated in cancer centres and 237 (74%) in general community hospitals (Table 1). Every participating physician had to include all his consecutive patients during 1 month (chosen by local investigators for each institution) between November 1995 and September 1996. Each patient was followed for two consecutive courses of chemotherapy.

#### Elypse 0 series

This series has already been described in the initial publication of the day 5 risk model (Blay *et al*, 1996). It comprises 329 patients evaluated in the test and validation samples of the previous publication, that is: (1) a retrospective series of 112 consecutive different patients treated in the Department of Intensive Chemotherapy of the Centre Léon Bérard between June and December 1992; (2) a prospective series of 142 patients treated in the Centre Leon Bérard between April and June 1993; (3) a prospective series of 36 patients treated with chemotherapy in the Institut Gustave Roussy between January 1994 and March 1994 and (4) a series of 39 patients treated with ACVBP within prospective protocols of the GELA group (Blay *et al*, 1996).

#### Chemotherapy regimens

Chemotherapy regimens were separated into two subgroups according to the doses of drugs administered. The first group, termed ‘high-risk’ regimens, includes highly cytotoxic regimens with a high expected incidence of neutropenia and FN as defined in previous works of the Elypse group (Blay *et al*, 1996,^1998^; Ray-Coquard *et al*, 1999,^2001^). High-risk regimens were defined on the basis of the initial recommendations of the French Ministry of Health for the use of G-CSF or GM-CSF, which was restricted to those patients receiving regimens including at least these doses of chemotherapy (for anthracyclines, alkylating agents, and VP16). For CDDP and cytosine arabinoside, the thresholds of doses chosen were those used internally, within the CLB, to select patients for primary prophylaxis with G-CSF or GM-CSF. ‘High-risk’ chemotherapy regimens are defined as follows: these are regimens containing doxorubicin or epirubicin ⩾90 mg m^−2^, or cisplatin ⩾100 mg m^−2^, or ifosfamide ⩾9 g m^−2^, or cyclophosphamide ⩾1 g m^−2^; or etoposide ⩾500 mg m^−2^, or cytarabine ⩾1g m^−2^ per course. The other subgroup includes all other chemotherapy regimens.

### Statistical analysis

Risk factors for FN were tested in univariate and multivariate analysis using the procedures of the SPSS® 10.0 program (SPSS, Inc., Chicago, 2000) on the CLB-1996 series of patients, in which only the haematological parameter measured at day 1 were available. The correlation between a clinical or a biological parameter and the incidence of chemotherapy-induced FN was performed using the *χ*^2^ test or the Fisher exact test. Logistic regression including the studied parameters of the univariate analysis was performed using the logistic program of SPSS 10.0®: a forward regression procedure was used with a *P* value <0.05 for entry. Risk factors (e.g. PS>1) and the end point (i.e. FN) were dichotomised. Parameter estimates for independent risk factors were determined and compared to previous risk model (Blay *et al*, 1996). This new risk model and the previous risk model (day 5 risk model) (Blay *et al*, 1996) were then compared in the Elypse 1 and 0 series. Finally, the sensitivity, specificity, positive predictive value, and negative predictive value (NPV) of the model were calculated. The relative risk of the high-risk group is estimated as the positive predictive value divided by 1-NPV.

## RESULTS

### Risk model for FN using day 1 blood cell count

The predictive value of day 1 blood cell count for the risk of FN was investigated in the CLB-1996 series, which includes 950 patients treated in 1996 in the Department of Medicine of the CLB. A total of 92 (9.7%) experienced an FN. In univariate analysis, ‘high-risk’ chemotherapy, PS>1, tumour type, and female sex were significantly correlated to the risk of FN (Table 1). Among day 1 haematological parameters, only day 1 lymphocytes ⩽700 *μ*l^−1^ were found marginally (*P*=0.052) correlated to the risk of FN in the CLB-1996 series (Table 2). Comorbidity, age, tumour stage, day 1 haemoglobin, platelet, and neutrophil count did not correlate to the risk of FN in the CLB-1996 series (Table 1 and Table 2). In the multivariate analysis, day 1 lymphocyte count and ‘high-risk’ chemotherapy were identified as the only two independent risk factors for FN in this series (Table 3

**Table 3:** Independent risk factors for FN in the CLB-1996 series

	***β***	**s.e.**	***χ*^2^**	***P***	**Odds ratio**	**95% CI**
Intercept	−2.5	0.114	300.9	<0.001		
High-risk chemotherapy	0.99	0.300	10.93	0.001	2.69	1.07–2.8
Day 1 lymphocytes ⩽700 *μ*l^−1^	0.56	0.246	5.08	0.02	1.75	1.49–4.8

). This enables one to delineate a risk model for FN in which day-1 lymphopenia <700 *μ*l^−1^ is used instead of day-5 lymphopenia used in the previous model (Blay *et al*, 1996) (Table 3). In this variant ‘day 1’ model, the observed incidence of FN in patients with 0, 1, and 2 of these two risk factors was found to be 8, 13, and 44%, respectively, in the CLB-1996 series (Table 4

**Table 4:** Incidence of FN (*N*, %) in the three subgroups according to the two risk models

	**ELYPSE 0 (*N*=329)**	**ELYPSE 1 (*N*=321)**	**CLB-1996 (*N*=950)**		
	**Model 1 (day 5)**	**Model 2 (day 1)^a^**	**Model 1 (day 5)**	**Model 2 (day 1)**	**Model 2 (day 1)**
In the whole group	90/329 (27%)	20/321 (6%)	92/950 (10%)		
Lymphocyte ⩽700 *μ*l^b^	71/146 (48%)	16/49 (33%)	5/31 (16%)	4/36 (11%)	27/204 (14%)
Two risk factors	56/82 (69%)	7/18 (61%)	5/11 (45%)	3/6 (50%)	4/9 (44%)
One risk factor	30/125 (24%)	40/111 (36%)	6/61 (10%)	5/48 (9%)	36/275 (13%)
No risk factor	4/122 (3%)	13/135 (9%)	9/242 (3.7%)	12/264 (4%)	52/666 (8%)

a Data on day 1 lymphocyte count were missing for 52 patients including 26 who experienced FN.

b On day 5 for model 1; on day 1 for model 2.

). The relative risk of FN in the high-risk group is 4.75 compared to the intermediate plus low-risk groups.

### Prognostic factors for FN in the Elypse 1 and 0 series

A total of 321 patients treated in community hospitals or regional cancer centres were included in the Elypse 1 study between November 1995 and September 1996. In all, 20 (6%) experienced FN after the first course of chemotherapy. In univariate analysis, parameters correlated to the risk of FN were slightly different from those observed in the CLB-1996 series: high-risk chemotherapy regimens, age, PS ⩾2, previous episodes of FN, tumour type, but not gender, stage, bone marrow involvement, number of previous chemotherapy cycles, were found correlated to the risk of FN (Table 1). Of note, age >60 was associated with a significantly higher incidence of FN in the Elypse 1 series, while the opposite was observed in the Elypse 0 series, probably because of the use of different regimens. Age was not an independent prognostic factor for FN in any series (not shown). Day 5 lymphocytes ⩽700 *μ*l^−1^ were the only biological parameter significantly correlated to the risk of FN in the Elypse 1 series. Although the incidence of FN in patients with low day 1 lymphocyte count was two-fold that of remaining patients, the difference was not significant (Table 2).

A logistic regression was performed to identify independent risk factors for FN in the Elypse 1 series: using logistic regression, the only two independent parameters were day 5 lymphocytes ⩽700 *μ*l^−1^ (*β*=1.07±0.51, *P*=0.034) and high-risk chemotherapy regimen (*β*=1.86±0.55, *P*<0.001), in agreement with our previously described day 5 risk model (Blay *et al*, 1996). The observed incidences of FN were nine of 242 (3.7%), six of 61 (10%), and five of 11 (45%), respectively, in patients with none, one, and both of the two risk factors of the day 5 risk model.

### Comparison of day 1 and day 5 model in the Elypse series

The ‘day 1’ risk model was then tested in the Elypse 1 and 0 series, and a subgroup of patients with a risk of FN>40% was identified in both series (Table 4). However, only 5% of all patients who experienced FN belonged to the high-risk group in the CLB-1996 series; this proportion was 15% in the Elypse 1 series and 12% in the Elypse 0 series (Blay *et al*, 1996). In contrast, 25 and 62% of the patients who experienced FN were in the high-risk group of the day 5 model in the Elypse 1 and 0 series, respectively. Patients with lymphocyte count >700 *μ*l^−1^ at day 1 and ⩽700 *μ*l^−1^ at day 5 were found to have a similar incidence of FN than patients with day-1 lymphocytes ⩽700 *μ*l^−1^ (Table 4). Among the three series, the positive predictive values of the day 1 and day 5 risk models were 42 and 66%, while the NPVs were 89 and 91%, respectively. The specificity of the day 1 and day 5 risk models were 98 and 94%; however, the sensibility of the day 1 model was lower than that of the day 5 model (8 *vs* 55%).

## DISCUSSION

FN is a frequent and life-threatening complication of cancer chemotherapy, which can be partially prevented by the prophylactic use of haematopoïetic growth factors (G-CSG or GM-CSF) or by a reduction of the dose of chemotherapy (The American Society of Clinical Oncology, 1994; Blay *et al*, 1995; Boogaerts *et al*, 1995; Bennett *et al*, 1999). Haematopoietic growth factors can be given in secondary prophylaxis, after a first episode of FN or as primary prophylaxis, that is, prior to any event. In the latter situation, the selection of patients at high risk for FN is necessary to reduce the risk of giving an unnecessary treatment with possible side effects and a significant cost. Several scientific oncology societies (The American Society of Clinical Oncology, 1994; Blay *et al*, 1995; Boogaerts *et al*, 1995; Bennett *et al*, 1999) have proposed guidelines for the prescription of haematopoietic growth factors: these factors should be given preferentially to patients with a ‘relatively high risk’ of FN in whom dose reduction could affect significantly the therapeutical goal, that is, response to chemotherapy or survival (The American Society of Clinical Oncology, 1994; Blay *et al*, 1995; Boogaerts *et al*, 1995; Bennett *et al*, 1999).

The quantification of the individual risk for a given patient remains uneasy in clinical practice. Although the dose of chemotherapy significantly increases the risk of FN, this complication may occur after almost any type of chemotherapy regimen (Miser *et al*, 1987; Tannock *et al*, 1988; Velasquez *et al*, 1988; Coiffier *et al*, 1989; Elias *et al*, 1989; Nichols *et al*, 1991). Individual parameters of the patient contribute, therefore, significantly to the risk of FN, in addition to the type and dose of chemotherapy. The identification of these individual risk factors is an important objective of future studies in this field (The American Society of Clinical Oncology, 1994; Blay *et al*, 1995; Bennett *et al*, 1999). However, a limited number of studies have attempted to identify these individual risk factors in a general population of cancer patients. In a previous study, we reported that the dose of chemotherapy and a rapid (day 5) lymphopenia (⩽700 *μ*l^−1^) following the administration of chemotherapy were independent risk factors for FN. This enabled us to identify a subgroup of patients with a high risk of FN (>40%) in a general population of cancer patients treated in comprehensive cancer centres (Blay *et al*, 1996).

The results presented here show that the previous model (day 5 model) is also efficient to identify a subgroup at high risk for FN among patients treated in community hospital with cytotoxic chemotherapy. The same risk factors for FN, that is high-risk chemotherapy and day 5 lymphocyte count ⩽700 *μ*l^−1^, had independent prognostic value in patients treated in cancer centres and in community hospitals, further supporting the validity of the model.

Two reasons prompted us to investigate an alternative risk model using day 1 blood cell count. First, in the Elypse 1 series, patients with day 1 lymphopenia ⩽700 *μ*l^−1^ were found to have a two-fold increase of the risk of FN as compared to the remaining patients, and similar observations were made in the previous Elypse 0 series (Blay *et al*, 1996). This parameter may have failed to reach significance in these series possibly because of an insufficient power of the study. Second, the requirement of haematological parameters measured at day 5 of chemotherapy in order to identify high-risk patients is not optimally practical for growth factor prescription or other prophylactic measures in an outpatient setting. It would therefore be useful to have a model enabling the identification of high-risk patients at day 1.

The analysis of risk factors for FN in the CLB-1996 population, which comprises 950 patients in whom only day 1 haematological parameters were available, showed that additional clinical and biological parameters had a significant predictive value for FN. Day 1 lymphopenia ⩽700 *μ*l^−1^ significantly correlated to the risk of FN in univariate analysis. Age <60 was not correlated to the risk of FN in the CLB-1996 series. In contrast, in the Elypse 0 series, which includes mainly patients treated within wards devoted to dose-intensive chemotherapy regimens, age >60 was associated with a reduced incidence of FN, because of the less frequent use of dose-intensive regimens in this subgroup of patients. These discrepancies illustrate the variability of risk factors for FN in different selected series of patients. It is important to note, however, that age was not an independent prognostic factor for any series in this study.

Indeed, using logistic regression, day 1 lymphopenia ⩽700 *μ*l^−1^ and the dose of chemotherapy remained the only two independent prognostic parameters in the CLB-1996 series in which day 5 blood count was not available. This enables one to distinguish patients with 0, 1 and both of these two risk factors with this ‘day 1’ risk model. In the latter group, 44% of the patients of the CLB-1996 series experienced FN. This variant risk model for FN (day 1 model) also identified groups of patients at high risk for FN in the Elypse 1 and 0 series, with an observed incidence of FN of 50 and 61%, respectively, confirming the validity of the day 1 model to predict FN.

However, the sensitivity of this day 1 model was found to be lower than that of the day 5 model, when compared in the Elypse 1 or Elypse 0 series. Indeed, a 40–50% of patients with lymphocyte count >700 *μ*l^−1^ will experience a drop under 700 *μ*l^−1^ at day 5, and these patients were found to be at similar risk for FN than patients with lymphocyte counts ⩽700 *μ*l^−1^ at day 1. Day 5 lymphopenia is a more sensitive criteria than day 1 lymphopenia to identify patients at risk for FN. The ‘day 5’ risk model is therefore more powerful to detect patients who will experience FN, in particular in cohorts receiving dose-intensive regimens. It is possible that the phenotypic analysis of depleted lymphocyte subtypes at day 1 may improve the sensitivity of the day 1 model: in a recently reported study, patients with both day 1 CD4+ and CD56+ lymphopenia were found to be at high risk for FN in a series of 226 patients (Blay *et al*, 2001). The capacity of these parameters to identify a higher proportion of patients who will experience FN is currently being tested prospectively.

Finally, it is noteworthy that both the day 1 and day 5 models were more ‘sensitive’ in the Elypse 0 series which includes a high proportion (43%) of ‘high-risk’ chemotherapy regimens, as compared to the Elypse 1 and CLB-1996, in which 8 and 10% of patients received high-risk regimens. In these latter series, in which a significantly higher proportion of all patients have one or no risk factors for FN, a lower proportion of FN occurs within the high-risk group. The models described here are therefore more useful in groups of patients in whom a high proportion receive ‘high-risk’ chemotherapy.

In conclusion, these results indicate that the previously described risk model for FN, relying on day 5 lymphopenia and the dose of chemotherapy, is efficient for the identification of patients who will experience FN in cohorts treated in community hospitals, although its sensitivity is lower than for patients treated in comprehensive cancer centre, because of the higher proportion of patients receiving high-risk chemotherapy. Day 1 lymphopenia identifies a small subgroup of patients at risk for FN among patients receiving high-risk regimens, but is much less sensitive than day 5 lymphopenia in all series tested.
